# Assessing the Knowledge and Attitudes of Breastfeeding During the COVID-19 Pandemic

**DOI:** 10.7759/cureus.54475

**Published:** 2024-02-19

**Authors:** Georgina C Tiarks, Beatrice C Thomas, Chasity O’Malley

**Affiliations:** 1 College of Allopathic Medicine, Nova Southeastern University Dr. Kiran C. Patel College of Allopathic Medicine, Ft. Lauderdale, USA; 2 Medical Education, Nova Southeastern University Dr. Kiran C. Patel College of Allopathic Medicine, Fort Lauderdale, USA; 3 Medical Education, Wright State University, Boonshoft School of Medicine, Dayton, USA

**Keywords:** breastfeeding immunology, breastfeeding during covid-19, breastfeeding knowledge, breastfeeding education, breastfeeding attitudes, covid-19 pandemic, immunology, breastfeeding

## Abstract

Background

It is generally accepted that breastfeeding is a practice that provides valuable health benefits to both mother and baby. However, the COVID-19 pandemic unveiled questions regarding the safety of breastfeeding, leading to potential hesitation among the public. Our study seeks to appreciate the public’s understanding and attitudes toward breastfeeding during the COVID-19 pandemic.

Methods

An anonymous survey was distributed online through social media platforms. Demographic information was collected with questions specifically analyzing the knowledge and attitude of each participant. Calculations were performed using Spearman’s rho correlations to determine significant differences.

Results

Thirty-nine women were recruited. The average overall knowledge score was 93% correct. 87% of participants believe breastfeeding is better than formula feeding, and 92% stated they would breastfeed their infant if they knew it could protect their child from COVID-19.

Discussion

The results of our study indicated that although overall knowledge of breastfeeding during the COVID-19 pandemic was well understood, higher levels of education may play a role in the depth of one’s knowledge. In addition, while participants were reporting high levels of education about breastfeeding from their healthcare providers, few were receiving data specific to the pandemic. The data gathered from this study may help to target future educational initiatives.

## Introduction

Breastfeeding is a practice that has been used by mothers for centuries to ensure adequate nutrition for their young [1]. Global research has established that breastfeeding not only delivers essential nutrients to infants but also has a significant impact on lifelong immunity [2]. Breast milk contains a variety of beneficial bioactive defense factors such as cytokines, growth factors, lactoferrin, and antibodies [3]. Breast milk also offers vast advantages over breast milk substitutes. Studies have shown that by exclusively breastfeeding for a minimum of 3 months, one can significantly reduce the risk of diarrhea, ear infection, and respiratory infection in infants [3]. This can drastically reduce infant morbidity and mortality rates worldwide. Similarly, in infant populations who were breastfed for up to 6 months, there was a significant reduction in the development of allergic diseases [3]. In addition to this, research has shown that infants who are breastfed for longer than 6 months have a higher IQ than those not breastfed, are less likely to become obese later in life, and are protected against developing type I diabetes as a child or type II diabetes later in life [4]. The advantages to breastfeeding are so limitless that both the World Health Organization (WHO) and the United Nations International Children’s Emergency Fund (UNICEF) state that breastfeeding may be an important step to improving health worldwide [4]. Overall, the protective impact that breastfeeding has on infants is immeasurable. Yet, these advantages are not only limited to the infant. Mothers who breastfeed may have a lower risk of heart disease, obesity, type II diabetes, ovarian cancer, and breast cancer [4]. These benefits resulted in The World Cancer Research Foundation issuing recommendations for breastfeeding due to the protective effects that have been exhibited [4].

When the novel SARS-CoV-2 virus, also known as coronavirus disease 2019 or COVID-19, began to dictate the world, questions arose as to the safety of breastfeeding for both mother and infant. As the pandemic spread, concerns about this age-old practice appeared globally, putting millions of children and mothers at risk. However, recent research has emerged providing answers to many of these previously unanswered questions. After numerous studies discovered that the COVID-19 virus is not present in breast milk, the WHO and US Center for Disease Control and Prevention (CDC) collectively concluded that despite the ongoing pandemic, mothers should continue to breastfeed their infants [5]. Even in cases where the mother is infected and unable to breastfeed, the WHO recommends using a mother’s expressed breast milk over substitutes [5]. To date, no cases have been reported showing transmission of the virus through breastfeeding [6,7]. This is due to the antiviral mechanisms of breastmilk inactivating the infectious virus [7]. While a mother cannot transmit the COVID-19 virus through breast milk, antibodies to the virus have been identified in breast milk for up to 5 months after infection [6,8]. Both IgA and IgG antibodies were detected in breast milk with a higher concentration of IgA [8]. Research even suggests that a protein in breast milk, lactoferrin, may be responsible for preventing the binding of the COVID-19 spike protein to the ACE2 receptor; thereby preventing infection and essentially neutralizing the threat [5]. Lactoferrin can also impact binding between the virus and heparan sulfate glycosaminoglycans (HSPG) effectively blocking additional host binding sites [5]. Furthermore, as COVID-19 vaccines have become more accessible worldwide, scientists have begun to study their short-term effects. Research has revealed that pregnant women who receive an approved vaccine may transfer vaccine-elicited SARS-CoV-2 antibodies to their infant through cord blood, breastmilk, and trans-placental [6,9]. These findings have led governing bodies to recommend vaccination to both pregnant and lactating women.

Nevertheless, despite all this recent evidence, skepticism remains evident. Our study seeks to better understand mothers’ current attitudes and knowledge of breastfeeding amidst the COVID-19 pandemic.

## Materials and methods

An anonymous survey was posted to social media platforms and distributed by investigators. Inclusion criteria specified that participants must be female, 18 years of age or older, who have either breastfed in the past 18 months, were currently breastfeeding, or were expecting to breastfeed in the next 9 months. The data collected did not contain any identifiable information and was recorded using the REDCap survey software. The survey included a total of 19 questions some of which had been modified from previously published breastfeeding questionnaires [1,10]. Twelve of these questions asked the participant which of the following statements best applied to them, while seven questions asked them to indicate whether they believed the following knowledge-based statements to be true or false. The information used in the true or false questions was gathered using peer-reviewed articles [1,5,9,10]. Demographic information (e.g., age group, ethnicity, education level) was also collected.

Descriptive statistics were used for demographic data. Spearman’s rho correlations were calculated using the IBM SPSS 28.0 statistics package.

The study was determined to be exempt by the Institutional Review Board at Nova Southeastern University (protocol 2021-347-NSU). Electronic informed consent was received by all individuals prior to their participation.

## Results

Demographics

The age ranges of the 39 participants included 7.7% comprising of 22 - 25 years old, 30.8% being in the 26 - 30-year age range, 43.5% being in the 31 - 35 years, 15.4 % being in the 36-40-year age range, and 2.6% comprising the 41- 45 years age range (Table 1). The educational levels consisted of 10.3% completing high school, 5.1% completed associate’s degrees, 41.0% completed bachelor’s degrees, and 43.6% completed graduate degrees (Table 1). The racial composition was mostly White/Caucasian (87.1%), with 2.6% each Asian, Black/African American, or other; and 5.1% Hispanic/Latino (Table 1). The majority of participants were exclusively breastfeeding (61.5%), with 33.3% breastfeeding and supplementing, 2.6% using only supplementation (not breastfeeding), and 2.6% using other methods (Table 1).

**Table 1:** Demographic Data

Demographic Category		Participant Distributions	
		Group	Percent (%)
1.	Age Range	22-25 years	7.7
		26-30 years	30.8
		31-35 years	43.5
		36-40 years	15.4
		41-45 years	2.6
2.	Educational Level	High school	10.3
		Associate’s degree	5.1
		Bachelor’s degree	41.0
		Graduate degree	43.6
3.	Race	Asian	2.6
		Black/African American	2.6
		Hispanic/Latino	5.1
		White/Caucasian	87.1
		Other	2.6
4.	Method for Feeding Child	Exclusively breastfeeding	61.5
		Breastfeeding with supplementation	33.3
		Feeding with supplements (not breastfeeding)	2.6
		Other	2.6

Knowledge-based questions

**Figure 1:**
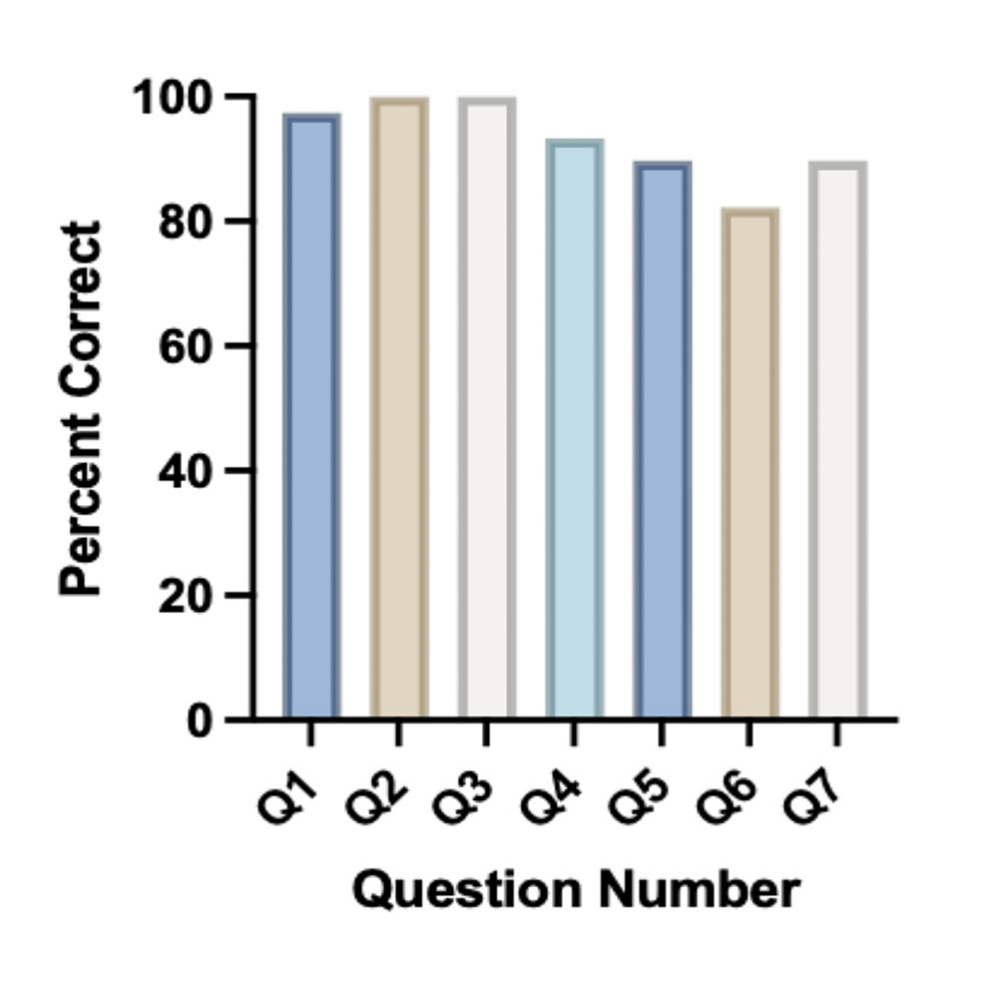
Average percent correct per question on knowledge-based information.

Overall, participants performed well on the knowledge questions. For Q1: The WHO does not recommend exclusively breastfeeding during the first 6 months of life. (False), the average score was 97.4% (Figure 1). For Q2: Breast milk may protect the baby from infectious diseases by transmitting antibodies to the infant. (True), and Q3: Breast milk may help with the development of an infant's immune system (Figure 1). (True), the percent correct was 100% (Figure 1). For Q4: Breast milk does not contain antibodies to COVID-19. (False), the percent correct was 93.3%. For Q5: You can pass antibodies from the COVID-19 vaccine through breast milk (True) and Q7: If a mother has tested positive for COVID-19, the CDC recommends that she continue to breastfeed (True), the percent correct for each was 89.7% (Figure 1). For Q6: You cannot transmit the COVID-19 virus through breast milk (True), the percent correct was 82.1% (Figure 1).

There was a moderate positive correlation between educational level and getting questions 4 (r=0.348, n=39, p=0.030) and 5 (r=0.473, n=39, p=0.002) correct (Table 2). Additionally, there was a moderate positive correlation between education level and the average score on all seven of the knowledge questions (r=0.398, n=39, p=0.012) (Table 2).

**Table 2:** Correlations Table *** indicates p=0.002; ** indicates p=0.012; * indicates p=0.030

			Education Level	Q1	Q4	Q5	Q6	Q7	Average Score
Spearman's rho	Degree Type	Correlation Coefficient	1.000	0.227	0.348^*^	0.473^***^	0.268	0.008	0.398**
		Sig. (2-tailed)		0.165	0.030	0.002	0.100	0.961	0.012
		N	39	39	39	39	39	39	39

**Figure 2:**
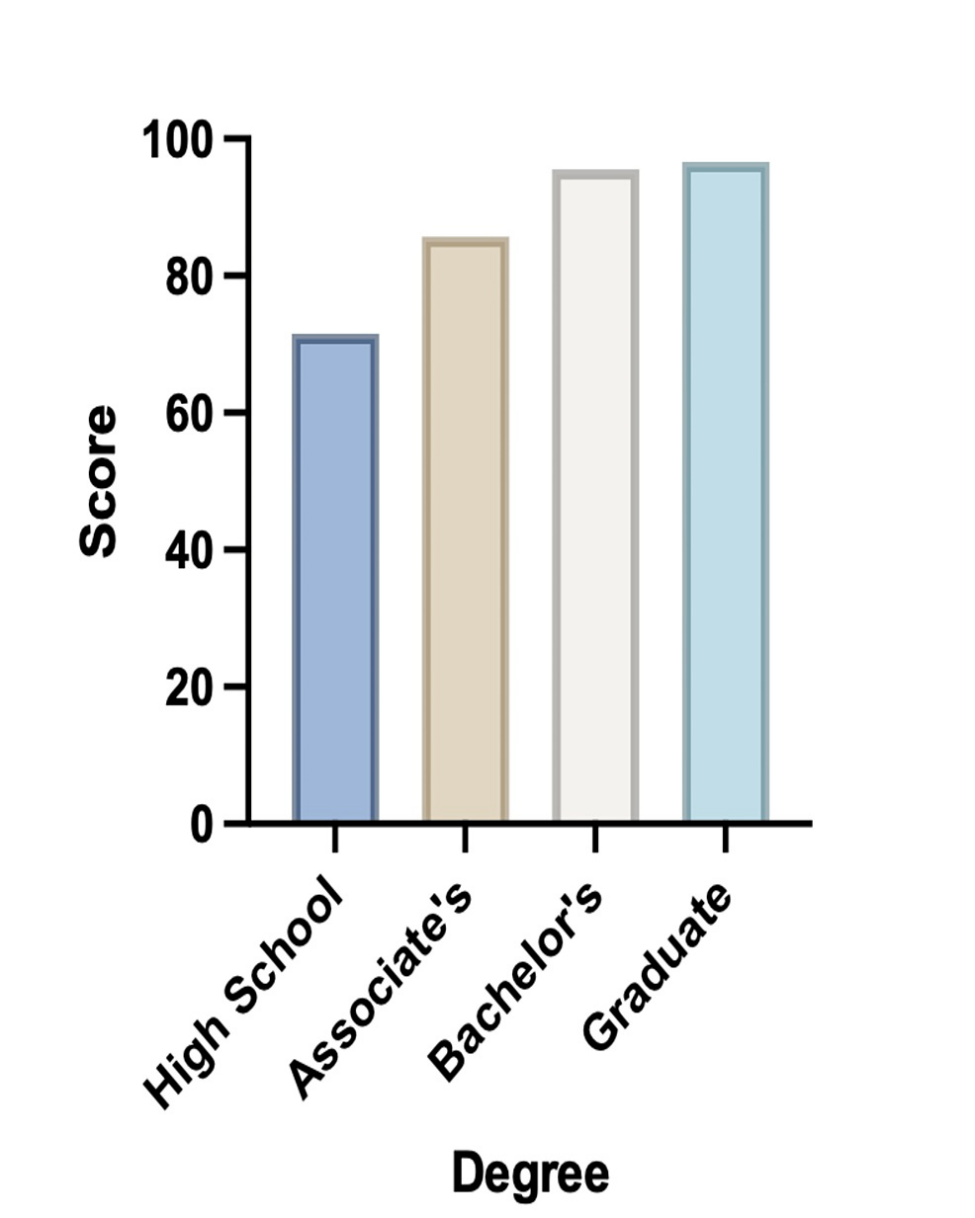
Average total score on knowledge-based questions based on educational level.

The average score overall for all groups was 93.0% (n=39) with High School graduates averaging 71.4% (n=4), people with associate's degrees averaging 85.7% (n=2), people with bachelor’s degrees averaging 95.5% (n=16), and people with graduate degrees averaging 96.6% (n=17) (Figure 2).

Attitude-based questions

**Figure 3:**
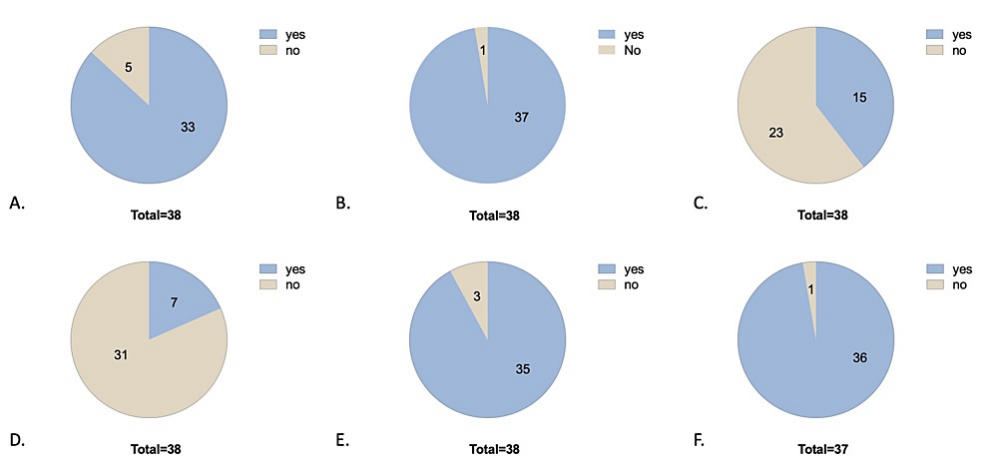
Demonstrates percent correct for attitude questions. Pie charts depicting respondents’ views regarding attitude-based questions including (3A) “Do you believe breastfeeding is better than formula feeding for your infant?”, (3B) “Did you receive information regarding breastfeeding from a healthcare professional?”, (3C) “Have you received any information about breastfeeding with regard to the COVID-19 pandemic from a healthcare professional?”, (3D) “Has your view of breastfeeding changed in light of the COVID-19 pandemic?”, (3E) “Would you be more likely to breastfeed if you knew you could protect your child from COVID-19?”, and (3F) “Are you planning to breastfeed or currently breastfeeding during the COVID-19 pandemic?”.

Attitudes related to breastfeeding were mostly positive toward breastfeeding. Ninety-seven percent (38 of 39) of participants had received any information about breastfeeding from a healthcare professional, while only 38% (15 of 39) received any information about breastfeeding with regard to the COVID-19 pandemic from a healthcare professional (Figures 3A-3B). Eight-seven percent (34 of 39) of participants believe breastfeeding is better than formula feeding for their infant, though 97% (37 of 39) of participants were planning to breastfeed or were currently breastfeeding during the COVID-19 pandemic (Figures 3C-3D). Twenty percent (7 of 39) of participants reported that their view on breastfeeding changed in light of the COVID-19 pandemic; however, 92% (36 of 39) reported that they would be more likely to breastfeed if they knew they could protect their child from COVID-19 (Figures 3E-3F).

## Discussion

The purpose of this study was to understand how women’s knowledge and attitudes toward breastfeeding had changed during the pandemic. The data gathered through this survey indicates that the population that we sampled was well-educated on topics regarding breastfeeding during the COVID-19 pandemic. This may have been partly due to the number of highly educated respondents, with more than 43% having a graduate degree, a quantity well over the national average. The majority of the knowledge-type questions were answered correctly. All questions asking whether breastmilk can protect a baby from infectious diseases through antibody transfer were answered correctly. Participants also correctly answered the question related to how breastmilk may improve an infant’s immune system development. The most incorrect question was whether the COVID-19 virus could be transmitted through breastmilk.

Based on the data, participants with higher education levels showed a higher level of knowledge in the questions given. In these groups, those who answered their highest level of education with a high school or associate’s degree had fewer knowledge-based questions answered correctly. In contrast, those with a bachelor’s or graduate degree had more correct answers. Individuals with a graduate degree had the most correct answers out of any group. This data indicates that education level may play a key role in the overall knowledge obtained about breastfeeding during the pandemic. These results support the findings of other global large-scale studies that have shown women with no formal education have worse breastfeeding indicators compared with those who have received higher levels of education [11]. Over the past few decades, a pattern emerged showing increases in exclusive breastfeeding and early initiation of breastfeeding with more maternal education [11]. Other studies in the United States, Europe, and Indonesia yielded similar results supporting the notion that higher maternal education positively impacted breastfeeding initiation [12-15]. This may be because individuals with higher education are more likely to be exposed to or seek out resources explaining the benefits of breastfeeding. Individuals with doctorate, master’s, or bachelor’s degrees may have increased access to resources to learn more about the COVID-19 pandemic. Furthermore, individuals with a foundational knowledge of health systems may have a greater ability to absorb and grasp concepts presented to them. During the COVID-19 pandemic, immunology and microbiology terms were constantly utilized. Without that basic background, individuals may have found detailed educational initiatives challenging to understand, affecting how they were addressed [16]. Strydhorst and Landrum observe that one individual's household knowledge may be completely unknown to others when referencing educational initiatives during the COVID-19 pandemic [16]. Therefore, it is essential to consider the audience and their foundational health systems knowledge when dispersing medical information to the public.

Analysis of the attitude questions indicated an overwhelmingly positive outlook on breastfeeding. Most participants believed breastfeeding was better than formula feeding and planned to breastfeed their infants. This aligns with previous studies showing that many mothers plan to breastfeed and are aware of the many benefits that it provides [17]. A fifth of individuals did state that their views on breastfeeding had changed during the pandemic, though. Whether their views were changed positively or negatively was not surveyed.

Finally, this study uncovered a discrepancy in the information that healthcare professionals dispersed. Many participants acknowledged having received information about breastfeeding from a healthcare professional. However, interestingly, only a little over a third of individuals stated that they had received targeted information about breastfeeding during the pandemic. This data parallels the response to the Zika virus. A similar study aimed to discover how much expectant mothers knew about the Zika virus. That study found that a little over a third of patients had been counseled specifically about Zika, leaving two-thirds of mothers uneducated and reliant on outside resources [18]. Although only some women received data about breastfeeding with COVID-19 from their healthcare professionals, they correctly answered the knowledge-based questions designed to test their insight. The data collected indicates that few healthcare professionals had shared information about the pandemic, which may have impacted mothers’ decision-making and led them to seek information elsewhere. This raises the question of where they were receiving their knowledge. A study was conducted in Belgium to understand the resources that women were using to gather data during pregnancy. While obstetricians were their primary resource, this was closely followed by the internet and pregnancy apps [19]. Additionally, a study conducted in the United States found that many mothers had used YouTube to learn about new recommendations for breastfeeding during the COVID-19 pandemic [20]. This study, among many others, found that healthcare professionals had created most of these videos to disperse information [20]. Yet, for there to be a demand for these YouTube videos, then their own healthcare professionals must not have been answering their questions adequately. Future public health initiatives may consider creating readily dispersible pamphlets, brochures, or websites for healthcare professionals to refer to so that they may be the primary source of medical information for their patients.

Limitations

This study has several limitations. There was a small sample size of people who participated in the surveys; therefore, the data gathered may not be representative of the larger population. Using surveys in data gathering also introduces the possibility of self-reporting bias. The anonymous surveys were distributed through social media by investigators. This included dispersal on breastfeeding-specific Facebook pages and outreach to the investigator’s communities. Individuals completing the survey may be composed of people seeking out breastfeeding information and those with higher levels of education.

## Conclusions

Overall, the results of this study show that public health initiatives to educate women on the use of breastfeeding during the pandemic were successful. There was a statistically significant difference in the knowledge responses based on degree type; however, with the small sample size of the population, the clinical significance is unknown. In the future, these results may support decisions to target groups with lower levels of education during health campaigns. Likewise, materials outlining health systems like immunology in layman’s terms may allow individuals to better understand the specialist terminology used to explain complex concepts.

Moreover, the results showed that although many women received information about breastfeeding from healthcare providers, few received pertinent COVID-19-related information. Women should receive more detailed information about breastfeeding from their healthcare providers, especially during pandemic outbreaks when mothers may have safety concerns. Public health officials may also want to consider the best ways to disperse this information, considering the limitations of quarantining during a pandemic. Future studies could also focus on assessing where pregnant women and women with infants receive breastfeeding guidance. Public health proposals could be aimed at improving resources for healthcare providers so that they are equipped with the knowledge and resources that they need to educate their patients on relevant public health emergencies.

## Appendices

Survey questions

Demographics

Please indicate which of the following applies best to you.

Please indicate your age in years:

·      18-21 years

·      22-25 years

·      26-30 years

·      31-35 years

·      36-40 years

·      41-45 years

·      46 years or older

What is your highest level of education?

·       High School

·       Associate’s Degree

·       Bachelor’s Degree

·       Graduate Degree

Which of the following best describes you?

·      White/Caucasian

·      Hispanic/Latino

·      Black/African American

·      Asian

·      Prefer not to say

·      Other

Please indicate your city and state (optional).

How are you currently feeding or planning to feed your baby?

·      Exclusive breastfeeding

·      Both breastfeeding and utilizing breast milk replacements

·      Feeding with breast-milk alternatives (not breastfeeding at all)

·      Other

Have you breastfed for previous pregnancies?

·      Yes

·      No

·      I have not had a previous pregnancy

Attitude

Please indicate which of the following applies best to you.

Have you received any information about breastfeeding from a healthcare professional?

·      Yes

·      No

Have you received any information about breastfeeding with regard to COVID-19 pandemic from a healthcare professional?

·      Yes

·      No

Are you planning to breastfeed or currently breastfeeding during the COVID-19 pandemic?

·      Yes

·      No

Do you believe breastfeeding is better than formula feeding for your infant?

·      Yes

·      No

Has your view on breastfeeding changed in light of the COVID-19 pandemic?

·      Yes

·      No

Would you be more likely to breastfeed if you knew you could protect your child from COVID-19?

·      Yes

·      No

Knowledge

Please indicate whether you think each statement is true or false for the following questions.

The World Health Organization (WHO) does not recommend exclusively breastfeeding during the first 6 months of life.

·      True

·      False

Breast milk may protect the baby from infectious diseases by transmitting antibodies to the infant.

·      True

·      False

Breast milk may help with the development of an infant’s immune system.

·      True

·      False

Breast milk does not contain antibodies to COVID-19.

·      True

·      False

You can pass antibodies from the COVID-19 vaccine through breast milk.

·      True

·      False

You cannot transmit the COVID-19 virus through breast milk.

·      True

·      False

If a mother has tested positive for COVID-19, the CDC recommends that she continue to breastfeed.

·      True

·      False

## References

[1] Cascone D, Tomassoni D, Napolitano F, Di Giuseppe G (2019). Evaluation of knowledge, attitudes, and practices about exclusive breastfeeding among women in Italy. Int J Environ Res Public Health.

[2] Hanson LA (1998). Breastfeeding provides passive and likely long-lasting active immunity. Ann Allergy Asthma Immunol.

[3] Turck D (2005). Breast feeding: health benefits for child and mother. (Article in French). Arch Pediatr.

[4] Binns C, Lee M, Low WY (2016). The long-term public health benefits of breastfeeding. Asia Pac J Public Health.

[5] Vassilopoulou E, Feketea G, Koumbi L, Mesiari C, Berghea EC, Konstantinou GN (2021). Breastfeeding and COVID-19: From nutrition to immunity. Front Immunol.

[6] Sakalidis VS, Perrella SL, Prosser SA, Geddes DT (2022). Breastfeeding in a COVID-19 world. Curr Opin Clin Nutr Metab Care.

[7] Pang Z, Hu R, Tian L (2022). Overview of breastfeeding under COVID-19 pandemic. Front Immunol.

[8] Pace RM, Williams JE, Järvinen KM (2021). Characterization of SARS-CoV-2 RNA, antibodies, and neutralizing capacity in milk produced by women with COVID-19. mBio.

[9] Collier AY, McMahan K, Yu J (2021). Immunogenicity of COVID-19 mRNA vaccines in pregnant and lactating women. JAMA.

[10] Latorre G, Martinelli D, Guida P, Masi E, De Benedictis R, Maggio L (2021). Impact of COVID-19 pandemic lockdown on exclusive breastfeeding in non-infected mothers. Int Breastfeed J.

[11] Neves PA, Barros AJ, Gatica-Domínguez G, Vaz JS, Baker P, Lutter CK (2021). Maternal education and equity in breastfeeding: Trends and patterns in 81 low- and middle-income countries between 2000 and 2019. Int J Equity Health.

[12] Laksono AD, Wulandari RD, Ibad M, Kusrini I (2021). The effects of mother's education on achieving exclusive breastfeeding in Indonesia. BMC Public Health.

[13] Li R, Darling N, Maurice E, Barker L, Grummer-Strawn LM (2005). Breastfeeding rates in the United States by characteristics of the child, mother, or family: The 2002 National Immunization Survey. Pediatrics.

[14] Foster SF, Vazquez C, Cubbin C, Nichols AR, Rickman RR, Widen EM (2023). Breastfeeding, socioeconomic status, and long-term postpartum weight retention. Int Breastfeed J.

[15] Sarki M, Parlesak A, Robertson A (2019). Comparison of national cross-sectional breast-feeding surveys by maternal education in Europe (2006-2016). Public Health Nutr.

[16] Strydhorst NA, Landrum AR (2022). Charting cognition: Mapping public understanding of COVID-19. Public Underst Sci.

[17] Beggs B, Koshy L, Neiterman E (2021). Women’s perceptions and experiences of breastfeeding: a scoping review of the literature. BMC Public Health.

[18] Guo F, Norton AR, Fuchs EL, Hirth JM, Garcia-Blanco MA, Berenson AB (2017). Provider-patient communication about Zika during prenatal visits. Prev Med Rep.

[19] Lanssens D, Thijs IM, Dreesen P (2022). Information resources among Flemish pregnant women: Cross-sectional study. JMIR Form Res.

[20] Azak M, Yılmaz B, Şahin N (2023). Analysis of YouTube© videos regarding breastfeeding during the coronavirus disease (COVID-19) pandemic. Matern Child Health J.

